# The morphospace of language networks

**DOI:** 10.1038/s41598-018-28820-0

**Published:** 2018-07-11

**Authors:** Luís F. Seoane, Ricard Solé

**Affiliations:** 1 Department of Physics, Massachusetts Institute of Technology, 02139 Cambridge, MA USA; 2 ICREA-Complex Systems Lab, Universitat Pompeu Fabra, 08003 Barcelona, Spain; 3 Institut de Biologia Evolutiva (CSIC-UPF), 08003 Barcelona, Spain; 4 Santa Fe Institute, 399 Hyde Park Road, Santa Fe, NM 87501 USA

## Abstract

What is the nature of language? How has it evolved in different species? Are there qualitative, well-defined classes of languages? Most studies of language evolution deal in a way or another with such theoretical contraption and explore the outcome of diverse forms of selection on the communication matrix that somewhat optimizes communication. This framework naturally introduces networks mediating the communicating agents, but no systematic analysis of the underlying landscape of possible language graphs has been developed. Here we present a detailed analysis of network properties on a generic model of a communication code, which reveals a rather complex and heterogeneous morphospace of language graphs. Additionally, we use curated data of English words to locate and evaluate real languages within this morphospace. Our findings indicate a surprisingly simple structure in human language unless particles with the ability of naming any other concept are introduced in the vocabulary. These results refine and for the first time complement with empirical data a lasting theoretical tradition around the framework of *least effort language*.

## Introduction

The origins of complex forms of communication, and of human language in particular, defines one of the most difficult problems for evolutionary biology^1–5^. Language makes our species a singular one, equipped with an extraordinary means of transferring and creating a virtually infinite repertoire of sentences. Such an achievement represents a major leap over genetic information and is a crucial component of our success as a species^6^. Language is a specially remarkable outcome of the evolution of cognitive complexity^7,8^ since it requires perceiving the external world in terms of objects and actions and name them using a set of signals.

Modelling language evolution is a challenging issue, given the unavoidable complexity of the problem and its multiple facets. Language evolution takes place in a given context involving ecological, genetic, cognitive, and cultural components. Moreover, language cannot be described as a separate collection of phonological, lexical, semantic, and syntactic features. All of them can be relevant and interact with each other. A fundamental issue of these studies has to do with language evolution and how to define a proper representation of language as an evolvable replicator^9^. Despite the obvious complexities and diverse potential strategies to tackle this problem, a common feature is shared by most modelling approximations: an underlying bipartite relationship between signals (words) used to refer to a set of object, concepts, or actions (meanings) that define the external world. Such mapping asumes the existence of speakers and listeners, and is used in models grounded in formal language theory^10^, evolutionary game theory^11,12^, agent modelling^13–17^, and connectionist systems^18^.

**Figure 1:**
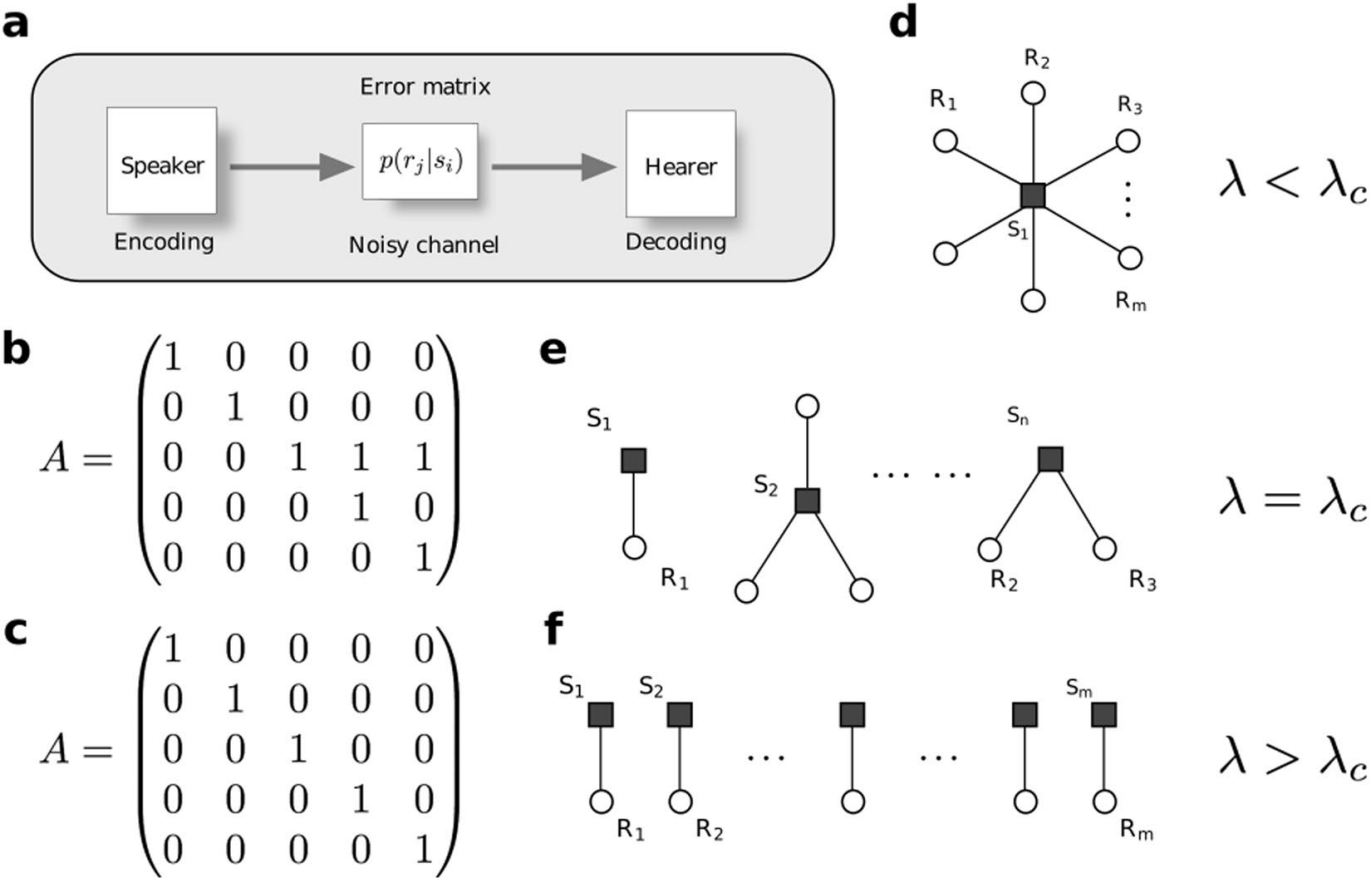
A toy model to explore *least effort language*. (**a**) Any minimal model of communication should include a (possibly noisy) channel that connects hearers and speakers. At the heart of this channel lies a confusion matrix *p*(*r*_*j*_|*s*_*i*_) that tells the likelihood that an object is interpreted by the hearer when a signal is uttered by the speaker. In an ideal, not noisy situation we can encode these word-object associations by a matrix (**b**,**c**) such that *a*_*ij*_ = 1 if signal *s*_*i*_ names object *r*_*j*_ and *a*_*ij*_ = 0 otherwise. Such matrices naturally introduce synonymy and polysemy. They also define bipartite *language networks* (**d**–**f**). We study how an optimization problem posed on the communication channel is reflected in optimal languages, with extreme solutions resulting in minimal effort for a speaker (hence maximal for a hearer, (**d**)) or the other way around (**f**).

In all these approaches, a fundamental formal model of language includes (Fig. 1a): i) a speaker that encodes the message, ii) a hearer that must decode it, and iii) a potentially noisy communication channel^19^ described by a set of probabilities of delivering the right output for a given signal. Within the theory of communication channels, key concepts such as reliability, optimality, or redundancy become highly relevant to the evolution of language.

In looking for universal rules pervading the architecture and evolution of communication systems, it is essential to consider models capable of capturing these very basic properties. Such a minimal toy model^20^ can be described as a set

1 $$S=\{{s}_{i},i=1,\ldots ,n\}$$

of available signals or “words”, each of which might or might not name one element from the set

2 $$R=\{{r}_{j},j=1,\ldots ,m\}$$

of objects or “meanings” existing in the world. These potential associations can be encoded by a matrix $A\equiv \{{a}_{ij}\}$ such that *a*_*ij*_ = 1 if signal *s*_*i*_ names object *r*_*j*_ and *a*_*ij*_ = 0 otherwise (Fig. 1e,f).

Following a conjecture by George Zipf^21^, this model was used to test whether human language properties could result from a simultaneous minimization of efforts between hearers and speakers^20^. Briefly, if a signal in language *A* names several objects in *R* a large decoding effort Ω_*h*_ falls upon the hearer (Fig. 1d shows a limit case of one signal naming every object). Otherwise, if one (and only one) different signal exists to name each of the elements in *R* (Fig. 1c,f), the burden Ω_*s*_ falls on the speaker who must find each precise name among all those existing, while decoding by the hearer is trivial. Minimal effort for one side implies maximal cost for the other. Zipf suggested that a compromise between these extremes would pervade the efficiency of human language.

This toy model allows us to quantify these costs and tackle the Zipfian *least effort* using information theory^20^. We do so by considering a linear ‘energy’ Ω(*λ*) containing both the hearer and speaker’s costs, which optimal languages would minimize:

3 $${\rm{\Omega }}(\lambda )=\lambda {{\rm{\Omega }}}_{h}+(1-\lambda ){{\rm{\Omega }}}_{s}.$$

*λ* ∈ [0, 1] is a metaparameter balancing the importance of both contributions. In terms of information theory, it is natural to encode Ω_*s*_ and Ω_*h*_ as entropies (see Methods). The global minimization of equation 3 was tackled numerically^20^ and analytically^22,23^. Slight variants of the global energy have also been studied, broadly reaching similar conclusions. An interesting finding is the presence of two “phases” associated to the extreme solutions just discussed (Fig. 1d,f). These opposed regimes were associated to rough representations of a “no-communication possible” scenario with one signal naming all objects (Fig. 1d), and a non-ambiguous (one-to-one, Fig. 1f) mapping associated to animal or computer languages. These phases are separated by an abrupt transition at a given critical value *λ*_*c*_. It was conjectured that human language would exist right at this transition point.

Solutions of this linear, single-targeted optimization have been found^22,23^. They display a mixture of properties, some associated (and some others not) to human language features. But: is the linear constraint a reasonable assumption? If no predefined coupling between Ω_*h*_ and Ω_*s*_ exists, the simultaneous optimization of both *targets* becomes a *Multi Objective* (or Pareto) *Optimization* (MOO)^24–27^. This more general approach does not make additional assumptions about the existence of global energies such as equation 3. MOO solutions are not single, global optima, but collections of designs (in this case, word-object associations encoded by matrices) constituting the optimal tradeoff between our optimization targets. This tradeoff (called the Pareto front) and its shape have been linked to thermodynamics, phase transitions, and criticality^24,28–31^.

The Pareto front for the MOO of language networks has never been portrayed. Here we aim at fully exploring the space of communication networks in the speaker/hearer effort space where the Pareto front defines one of its boundaries (see Methods). We will further study the whole space of language networks beyond the front, illustrating the wealth of communication codes embodied by all different binary matrices. These, as they link signals and objects, naturally define graphs with information about how easy communication is, how words relate to each other, or how objects become linked in semantic webs. All these characteristics pose interesting, alternative driving forces that may be optimized near the Pareto front or, in the contrary, pull actual communication systems away from it.

Our exploration defines a *morphospace* of language networks. The concept of *theoretical morphospace*^32^ was introduced within evolutionary biology^33–35^ as a systematic way of exploring all possible structures allowed to occur in a given system. This includes real (morphological) structures as well as those resulting from theoretical or computational models. Typically a morphospace is constructed in one of two different ways. From real sets of data, available morphological traits are measured and some statistical clustering method (e.g. principal components) is applied to define the main axes of the space and locate each system within it^32^. Alternatively, explicit parameters define continuous axes that allow ordering all systems in a properly defined metric space. In recent years, graph morphospaces have been explored showing how morphospaces can be generalized to analyze complex networks^36^. Our language morphospace is shown to be unexpectedly rich. It appears partitioned into a finite set of language networks, thus suggesting archetypal classes involving distinct type of communication graphs.

Finally, dedicated, data-driven studies exist about different optimality aspects of language, from prosody to syntax among many others^37–41^. Discussion of the least-effort language model above has focused on its information theoretical characterization. The hypothesis that human language falls near the phase transition of the model has never been tested on empirical data before. We do so here using the WordNet database^42,43^. Our development of the morphospace allows us not only to asses the optimality of real corpora, but also to portray some of its complex characteristics. This kind of study may become relevant for future evolutionary studies of communication systems, most of them relying on the “speaker to noisy-channel to hearer” scheme (Fig. 1) at the core of the least effort model.

## Results

**Figure 2:**
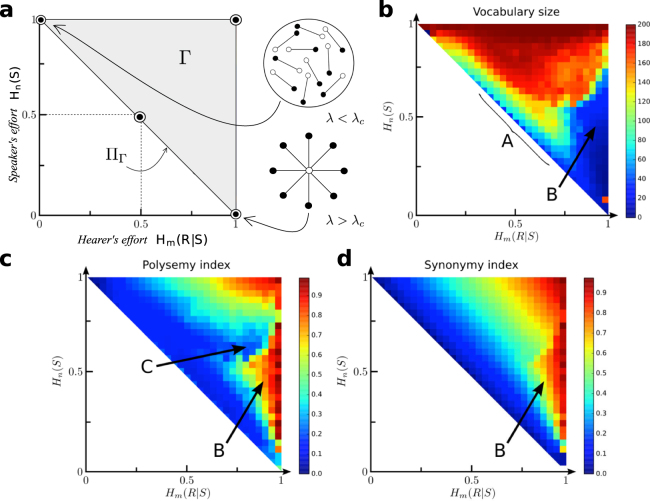
Vocabulary size, polysemy, and synonymy across language morphospace. (**a**) The space Γ that can be occupied by language networks is shown in gray. Two limit cases (the one-to-one and star graphs) are also mapped. (**b**) Effective vocabulary size is only low near the star graph (in a prominent area labeled B) and along the Pareto front. (**c**) Polysemy is large in region B and as we complete the matrix *A* towards the upper-right corner. (**d**) Synonymy increases uniformly as we move apart from the front except for codes in B. This makes them highly Pareto inefficient.

In the Methods section we define the *design space* Γ of the least-effort model (i.e. the set of possible languages within the model) and show where it lives within the *language morphospace*. The morphospace has as axes the MOO target functions (i.e. the costs Ω_*h*_, Ω_*s*_ associated to hearers and speakers, Fig. 2a). We sampled this space and performed measurements upon the language networks found (as explained in Methods). Thus we capture information such as a language’s degree of synonymy, how well its word distribution fits Zipf’s law, etc. This section reports the main findings after projecting these measurements onto the morphospace. Similar results are reported for Pareto optimal languages alone (Appendix A).

### Complexity of language morphospace

Figure 2 shows the boundaries of our morphospace (see Methods) and the location of some prominent solutions: i) the star graph, which minimizes the effort of a speaker and maximizes that of a hearer; ii) the one-to-one mapping, often associated to animal communication, which minimizes the effort of a hearer at the expense of a speaker’s; and iii) the Pareto optimal manifold (Π_Γ_) corresponding to the lower, diagonal boundary of Γ in the Ω_*h*_ − Ω_*s*_ plane. Π_Γ_ tells us the optimal trade-off between both targets.

#### Characterizing the vocabulary

The effective vocabulary size *L* (equation 13) across the morphospace reveals a non-trivial structure. Codes with small *L* occur mostly near the star and in a narrow region adjacent to the Pareto front (marked A in Fig. 2b). Far apart from the front there is yet another region (marked B) with languages that use ~30% of all available signals. The transition to codes that use more than ~75% of available signals (central, red region in Fig. 2b) appears abrupt wherever we approach those codes from.

The low-vocabulary region B consists mostly of very polysemic signals (Fig. 2c). But codes with small vocabularies are not always outstandingly polysemic – e.g. along the Pareto front. Right next to region B, the polysemy index *I*_*P*_ (equation 14) drops suddenly (area C in Fig. 2c) and then increases steadily as we tend towards the top right corner of Γ (where a matrix sits with ${a}_{ij}=1\forall i,j$).

Region B extends upwards from the star. It is also associated to a large synonymy index (*I*_*S*_, equation 15, Fig. 2d). This implies that *I*_*S*_ increases sharply around the star as codes become less Pareto optimal. This swift increase does not happen if we start off anywhere else from the front. The condition for Pareto optimality is that codes do not have synonyms (see Methods). This plot indicates that Pareto optimality degrades almost uniformly anywhere but near the star. This might have evolutionary implications: Languages around the B region require more contextual information to be disambiguated. That part of the morphospace might be difficult to reach or unstable if Pareto selective forces are at play.

#### Network structure

Words are not isolated entities within human language. Word inventories are only a first layer of complexity. To understand language structure we need to consider how linguistic units interact. There are diverse ways to link words together into graphs^44,45^, and it was early found that such language networks are heterogeneous (the distribution of links displays very broad tails) and highly efficient regarding navigation^46^. A network approach allows us to look at language from a system-level perspective, beyond the statistics associated to signal inventories. Even the toy model studied here has been used to gain insight into the origins of complex linguistic features such as grammar and syntax^47–49^).

**Figure 3:**
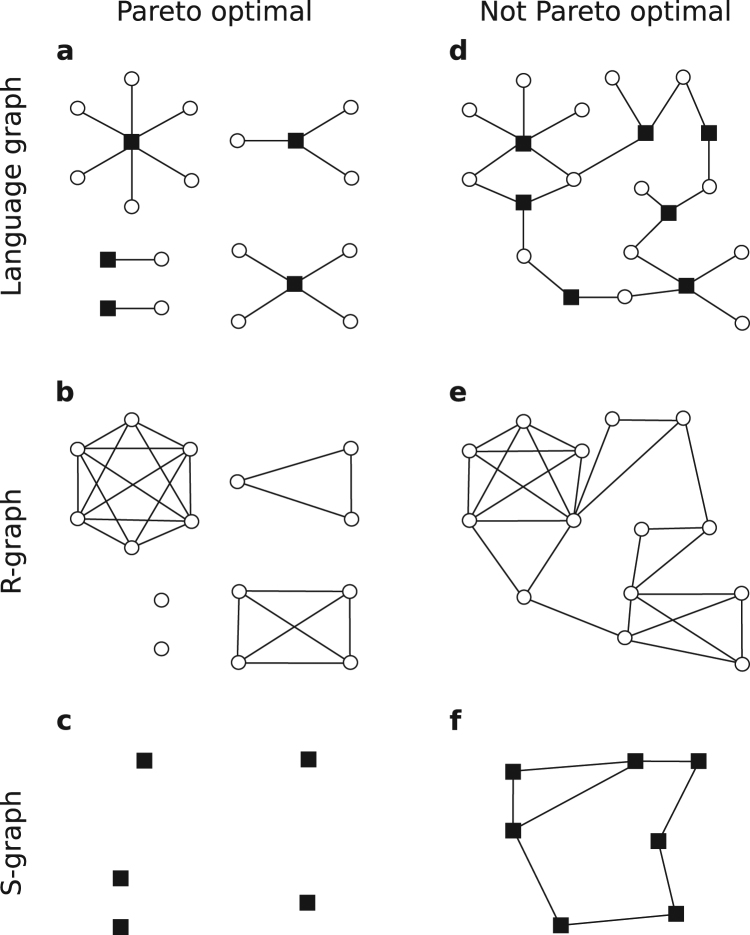
Different graphs derived from the language matrix. (**a**) A Pareto optimal language contains non-synonymous signals only. Its language graph consists of isolated clusters in which each signal clusters together a series of objects. (**b**) Concepts within a cluster appear as cliques in the *R*-graph. (**c**) The *S*-graph is just a collection of isolated nodes. (**d**) Not Pareto optimal languages produce more interesting language graphs that might be connected (as here) or not. A connected language graph guarantees both a connected *R*- and *S*-graphs ((**e**,**f**) respectively).

Our model defines three networks (see Methods for details). A first one (termed *code graph*) connects signals to objects (Figs 1d–f and 3a,d). Another one (termed *R*-*graphs*) connects objects to each other (Fig. 3b,e). Yet another one (termed *S*-*graph*) connects signals to each other (Fig. 3c,f).

**Figure 4:**
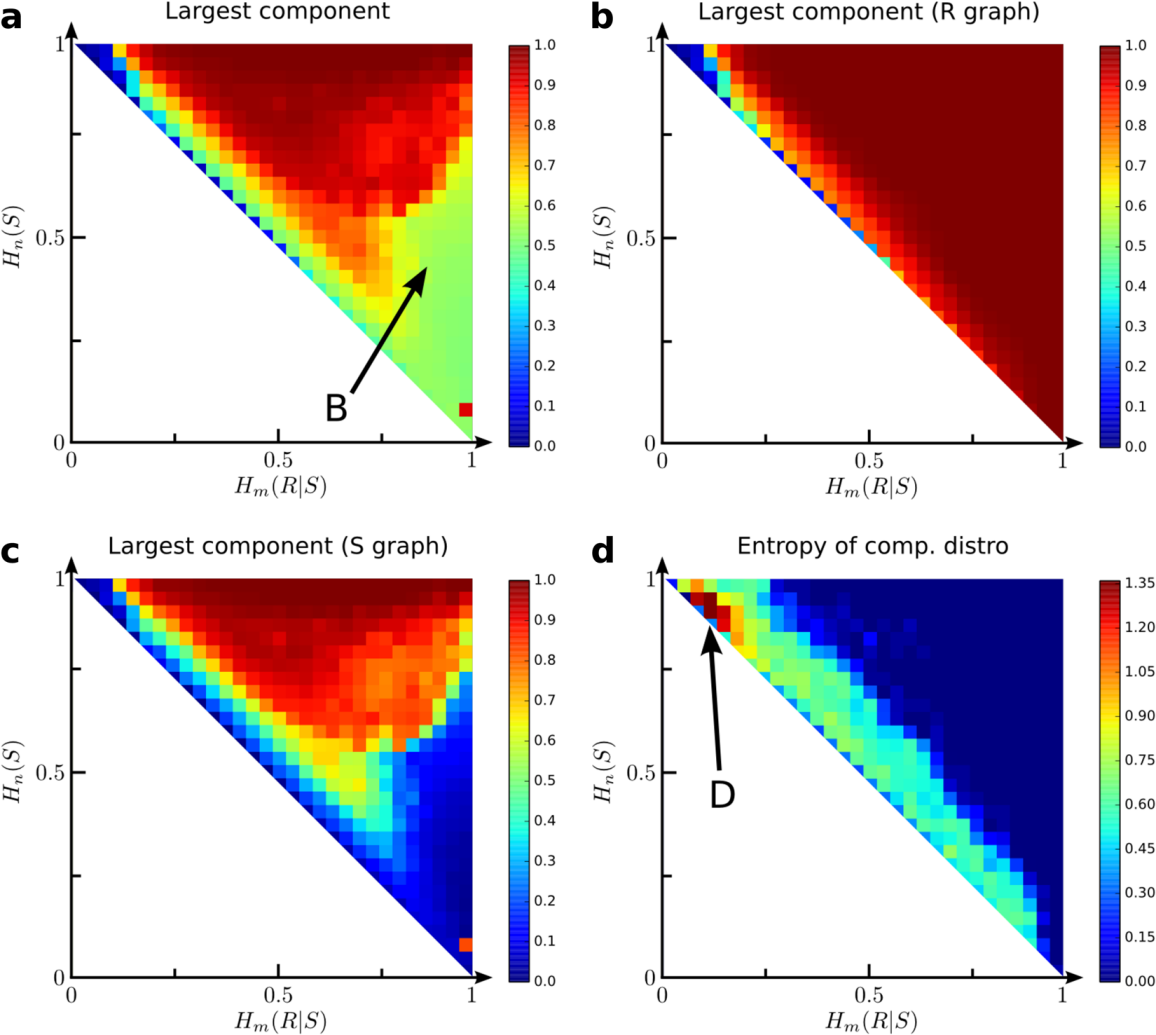
Network connectivity across the morphospace. The size of the largest connected component is shown for code graphs (**a**), *R*-graphs (**b**), and *S*–*graphs*. (**d**) Entropy of component size distribution is large around a band that runs parallel along the Pareto front.

The size of each network’s largest connected component (*C*_*max*_, equation 18) is shown in Fig. 4a–c for code graphs, *R*-graphs, and *S*-graphs. Code graphs with large *C*_*max*_ (Fig. 4a) widely overlap with large effective vocabularies (*L*, Fig. 2b). The B region is the exception: it displays moderate *C*_*max*_ values yet very low *L*. This *C*_*max*_ vanishes for *S*-graphs in the B region, but the corresponding *R*-graphs remain very well connected. Hence, in B a few signals keep together most of object space. Actually, *R*-graphs appear well connected throughout most of the morphospace (Fig. 4c), except in a narrow region extending from the one-to-one mapping along the Pareto front, more than halfway through it.

The entropy of connected components size distributions (*H*_*C*_, equation 19) somehow captures the heterogeneity of a language network. It is shown in Fig. 4d for code graphs (and is similar for *R*- and *S*-graphs). *H*_*C*_ is small everywhere except on a broad band parallel to the Pareto front. *H*_*C*_ is so low almost everywhere because of either of these facts: i) Only one connected component exists, as in most of the area with large vocabulary. ii) A few signals make up the network, deeming all others irrelevant. Effectively, all network features are summarized by a few archetypal graphs. iii) While a lot of signals are involved, they produce just a few different graphs. That shall be the case along the Pareto front (see Appendix A.2).

The band with larger *H*_*C*_ runs parallel to the front, a little bit inside the morphospace. Hence, if the heterogeneity of the underlaying network were a trait selected for by human languages, they would be pulled off the Pareto front. Finally, *H*_*C*_ is largest around region (D, Fig. 4d) close to the one-to-one mapping.

#### Complexity from codes as a semantic network

Language ties together real-world concepts into an abstract semantic web whose structure shall be imprinted into our brains^50,51^. It is often speculated that semantic networks must be easy to navigate. This in turn relates to small-world underlying structures^46,52^ and other system-level network properties. It would be interesting to quantify this using our language graphs as a generative toy model.

**Figure 5:**
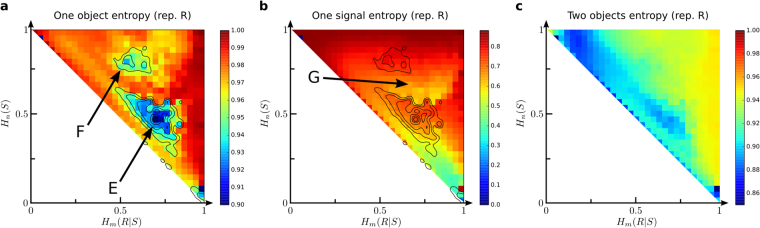
Complexity of codes as a random generative model. (**a**) Entropy of objects as sampled by a random walker (*H*_*R*_) over the language network is close to its maximum throughout the morphospace, except for two non-trivial areas labeled E and F. Whichever mechanisms give rise to the heterogeneity there, they seem to be different, since the transition between E and F is not smooth. (**b**) Entropy of signals as sampled by a random walker (*H*_*S*_) is lower than its maximum across the morphospace, and the most singular areas do not correlate with the ones found for (*H*_*R*_). Notably, region G seems to separate E and F and contain more heterogeneous signal sampling despite the largely homogeneous object sampling. (**c**) 2-grams of objects as sampled by a random walked present a lower entropy *H*_2*R*_ than *H*_*R*_, and only the E region seems to remain in place.

We did just so (see Methods), and we introduce a couple of entropies (*H*_*R*_, equation 20; and *H*_*S*_, equation 21) that capture the bias in sampling objects and signals with this generative toy model. These measures present non-trivial profiles across the morphospace. *H*_*R*_ drops in two regions (E and F in Fig. 5a). Code graphs around these areas must have some canalizing properties that break the symmetry between sampled objects. However, the drop in entropy is of around a 10% at most. (A third region with low *H*_*R*_ near the star graph is discussed in Appendix A.3).

From Figs 2b and 4a, region *E* has moderately large *L* and *C*_*max*_. It sits at a transition from lower values of these quantities (found towards the front and within the B region) to larger values found deeper inside the morphospace. Figure 4d locates region E right out of the broad band with large *H*_*C*_. All of this suggests that, within E, diverse networks of smaller size get connected into a large component which inherits some structural heterogeneity. This results in a bias in the sampling of objects, but not in the sampling of signals: the lowest *H*_*S*_ are registered towards the star-graph instead (see Appendix A.3). Note also that biases in signal sampling are larger (meaning lower *H*_*S*_) throughout the morphospace – compare color bar ranges in Fig. 5a,b.

Region F sits deeper inside the morphospace, where *L* is almost the largest possible and the connected component involves most of signals and objects. Language networks here are well consolidated, suggesting that the bias of object sampling comes from non-trivial topologies with redundant paths. Interestingly, regions E and F are separated by an area (G, Fig. 5b) with a more homogeneous sampling of objects and a relatively heterogeneous sampling of signals. *H*_*S*_ within F itself is larger than in G, suggesting no remarkable bias on word sampling in F despite the bias on object sampling, and vice-versa. The diversity found in the morphospace, which allows an important asymmetry between words and objects inducing heterogeneity in one set while keeping the other homogeneous.

Figure 5c shows *H*_2*R*_, the entropy of 2-grams objects produced by the generative toy model (see Methods). *H*_2*R*_ inherits a faded version of region E. On top, it is low along a band overlapping with the one in Fig. 4d for *H*_*C*_. The largest drop in *H*_2*R*_ happens closer to the one-to-one mapping. It makes intuitive sense that codes in this last area start consisting of networks similar to the one-to-one mapping in which extra words connect formerly isolated objects, hence resulting in a bias of couples of objects that appear together. The entropy of 2-gram words (*H*_2*S*_, not shown) is largely similar to *H*_*S*_ (Fig. 5b).

#### Zipf, and other power laws

Zipf’s law is perhaps the most notable statistical patterns in human language^21^. Despite important efforts^53–55^, the reasons why natural language should converge towards this word frequency distribution are far from definitive. Research on diverse written corpora suggests that under certain circumstances (e.g. learning children, military jargon, impaired cognition) word frequencies present pow er-law distributions with generalized exponents^56,57^.

Different authors have studied how well the least-effort toy model accounts for Zipf’s law^20,22,23^. Word frequencies can be obtained from the language matrices *A* (see Methods). The first explorations of the model^20^ found Zipf’s distributions just at the transition between the star and one-to-one codes. This suggested that self-organization of human language at the least-effort transition point could drive the emergence of Zipf’s distribution. It was shown analytically that while Zipf’s law can be found at that point, this is not the most frequent distribution^22,23^. This is consistent with the diversity that we find at the Pareto front (see Appendix A). This also implies that if least-effort is a driving force of language evolution, it would not be enough to constrain the word distribution to be Zipfian. Other authors^58^ have provided mathematical arguments to expect that Zipf’s law will be found right at the center of our Pareto front (with Ω_*h*_ = 1/2 = Ω_*s*_).

**Figure 6:**
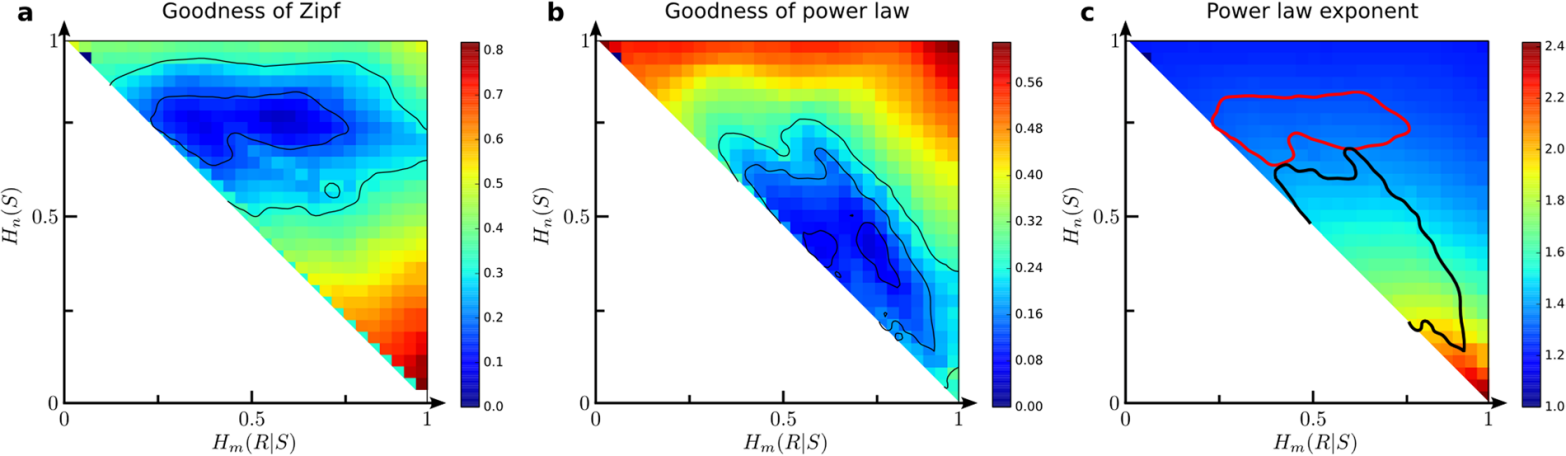
Power laws from the least-effort model. (**a**) Goodness of fit of the word distribution from the toy, least-effort model to a Zipf law. (**b**) Goodness of fit of the word distribution from the model to an arbitrary power law. (**c**) Exponent obtained when fitting the word distribution of the model to the arbitrary power law from panel (b). In each case, the level curves indicate areas where a Kolmogorov- Smirnov test suggest a good fit.

We compare signal distributions to Zipf and other power laws (see Methods). The area that better fits Zipf is broad and stretches notably inside the morphospace (Fig. 6a), hence Zipf’s law does not necessarily correlate with least-effort. This area runs horizontally with ${{\rm{\Omega }}}_{s}\equiv {H}_{n}(S)\sim 0.75$ and roughly ${{\rm{\Omega }}}_{h}\equiv {H}_{m}(R|S)\in (0.25,0.75)$. In the best (least-effort) case, speakers incur in costs three times higher than hearers. Less Pareto optimal Zipfian codes always attach a greater cost to speakers too.

Figure 6b shows how well distributions are fitted to arbitrary power laws. An alternative region with good fits runs parallel along the lower part of the Pareto front, but the corresponding power law exponents (Fig. 6c) fall around 1.6–1.8, far from Zipf’s.

These findings present notable evidence against least-effort as an explanation of Zipf’s law. Non Pareto-optimal codes exist with larger fitness to Zipf than least-effort languages (Fig. 6a), and codes along the Pareto front seem better fitted by other power laws (see Appendix A.4). Two important limitations of the model should be considered: First, the naive way in which word frequencies are built from the model (see Methods). Second, we examined relatively small matrices (200 × 200) to make computations tractable. Measuring power law exponents demands larger networks. Alleviating these handicaps of the model shall bring back evidence supporting the least-effort principle.

### Code archetypes and real languages

**Figure 7:**
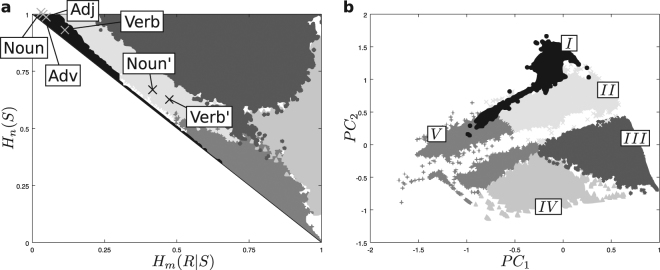
Clustering of languages across the morphospace. *k*-means clustering using all principal components reveals a consistent structure in the morphospace. Five cluster are shown here. Real languages fall within cluster *I*, close to the one-to-one mapping proper of animal communication systems. The real matrices are marked: *Adj* for the adjectives, *Adv* for the adverbs, *Noun* for the nouns, and *Verb* for the verb. If certain grammatical words are included (named with an apostrophe: *Noun*’ for nouns and *Verb*’ for verbs) they move into cluster *II* and towards the center of the morphospace, relatively close to the Pareto front. (**b**) All clusters get further segregated in two principal component space. This space appears interrupted by a stripe along which no codes exist.

We introduced different measurements over the matrices *A* of our toy model. The emerging picture, far from a smooth landscape, is a language morphospace that breaks into finite, non-trivial “archetypes”. We ran additional analyses to support this. We computed Principal Components (PCs) from all the measurements discussed. 5 PCs were needed to explain 90% of the data variability. We then applied a *k*-means algorithm^59^ on PC space. For *k* = 5, several runs of the algorithm converged consistently upon similar clusters that we classify as follows (Fig. 7, clockwise from top-left):

I Codes near the one-to-one mapping and upper two thirds of the Pareto front, including the graphs with largest *H*_*C*_ (Fig. 4d).
II Codes along a stripe parallel to the upper half of the Pareto front, overlapping largely with the large *H*_*C*_ (Fig. 4d) and low *H*_2*R*_ (Fig. 5c) area.
III Bulk interior region: codes with a single connected component, large vocabulary; includes low *H*_*R*_ region F (Fig. 5a).
IV Region B (Fig. 2b–d): codes with large polysemy, small vocabularies; demands exhaustive contextual cues for communication.
V Codes along the lower half of the Pareto front and a thick stripe parallel to it, partly overlapping with the region with good fit to power-laws (Fig. 6b).

Solutions to the original least-effort problem were widely analyzed in the literature from a theoretical viewpoint, focusing on the model’s phase transition^20^, on the presence of Zipf’s distribution at the transition point^20,22,23,46^, or on mechanisms that could drive languages to this distribution^30,56,58^. Based on these analyses it was speculated that human language should lie at the transition point to achieve its flexibility and expressive power. The one-to-one mapping, associated to animal codes, was deemed rather rigid and memory demanding. This raised a point that ambiguity would be the price to pay for least-effort efficient language. On the other hand, the star code makes communication impossible unless all the information is explicit in the context.

This toy model has never been used to assess real languages, perhaps, owing to the difficulty of building matrices *A* out of linguistic corpora. WordNet^42,43^ is a huge database that includes manually annotated relationships between words and objects or concepts. A few examples:

ape (…) 02470325 09964411 09796185

car (…) 02958343 02959942 02960501 …

complexity (…) 04766275

rugby (…) 00470966

The parentheses stand for additional information not relevant here. Each word is associated to several codes that identify a unique, unambiguous object or concept. For example, 02959942 refers to the car of a railway. 02960501 refers to the gondola of a funicular. The word “car” appears associated to these two meanings among others. WordNet makes this information available for four separate grammatical categories: adjectives, adverbs, nouns, and names.

We built the corresponding *A* matrices out of this database and evaluated *H*_*m*_(*R*|*S*) and *H*_*n*_(*S*) for each grammatical category. All four categories contain more signals than objects, hence synonyms exist and languages are not Pareto optimal. Theoretical models (including others beside ours) argue that synonyms should not exist in optimal codes^11,12,20,23^, but they seem real in folk language. Synonymy shall also have degrees, with linguists dissenting about whether two terms name the precise same concept. Such information is lost due to our coarse graining into binary matrices. It is possible to extend our analysis if *A* would display likelihoods ${a}_{ij}\in [0,1]$ indicating affinity between words and meanings.

Figure 7a shows all available grammatical categories (labeled *Adj*, *Adv*, *Noun*, and *Verb*) in our morphospace. While not Pareto optimal, they appear fairly close to the front, near the one-to-one mapping. This would suggest that human language is not such a great departure from animal codes, thus contradicting several arguments in least-effort literature. Also, all categories appear within a small area, leaving the huge morphospace unexplored.

The WordNet database does not contain grammatical words such as pronouns. Some proper names appear in the *Noun* database (e.g. Ada and Darwin), but ‘she’, ‘he’, or ‘it’ are not included. Any feminine proper name can be substituted by ‘she’, while ‘it’ can represent any common noun. Similarly, in English most verbs can be substituted by ‘to do’ or ‘to be’ – e.g. “She plays rugby!” becomes “Does she play rugby?” and eventually “She does!”. Appending such words to the database would account for introducing signals that can name almost any object. We simulated this by adding a whole row of 1s to the *A* matrices of nouns and verbs. This changed the corresponding *H*_*m*_(*R*|*S*) and *H*_*n*_(*S*) values, displacing these codes right into the central-lower part of cluster *II* (Fig. 7a, marked *Noun*’ and *Verb*’), near the center of the Pareto front. This suggests that grammatical words might bear all the weight in opening up the morphospace for human languages, with most semantic words conforming a not-so-outstanding network close to the one-to-one mapping and still demanding huge memory usage.

## Discussion

The least-effort model discussed in this paper has long captured the attention of the community. It features a core element of most communication studies – namely, the “coder to noisy-channel to decoder” structure found in Shannon’s original paper on information theory^60^, as well as in more recent experiments on the evolution of languages^13,15,16^. This toy model allows us to formulate several questions regarding the optimality of human language and other communication systems. These had been partly addressed numerically^20^ and analytically^22,23^. A first order phase transition was found separating the one-to-one mapping from a fully degenerated code. It was further speculated that human language may be better described by codes at that transition point^20^. This hypothesis was never confronted with empirical data. Finally, by looking only at least-effort languages a vast code morphospace was left unexplored.

This paper relies on Pareto optimality to recover the first order phase transition of the model^24,28,30^ and thus find the boundaries of the morphospace. We then characterize the very rich landscape of communication codes beyond the optimality constraints. Finally, we address for the first time empirically the hypotheses about the optimality of human language within the least-effort model.

This landscape turns out to be surprisingly rich, far from a monotonous variation of language features. Quantities such as the synonymy of a code, its network structure, or its ability to serve as a good model for human language (e.g. by owing Zipf’s law) present non-trivial variations across the morphospace. These quantities might or might not align with each other or with gradients towards optimality, and may hence pose newer conflicting forces that communication systems shall be shaped by.

To portray human languages within the least-effort formalism we resorted to the WordNet database^42,43^. Raw matrices extracted from this curated directory fell close to the one-to-one mapping (often associated to animal codes) and in the interior of the morphospace. This would invalidate previous hypotheses that human language belongs far apart from animal communication and along the transition point of the model. Introducing grammatical particles such as the pronoun ‘it’ (which can name any object and is missing from WordNet) moves human language far away from one-to-one mappings and closer to the center of the Pareto optimal manifold. Both found locations for human languages (before and after adding grammatical particles) present interesting properties such as a large entropy of concept-cluster size (*H*_*C*_, Fig. 4d). This quantity, which somehow captures the language network heterogeneity, drops to zero at the Pareto front, suggesting evolutionary forces that could pull real languages away from the least-effort optimality studied here.

Our results suggest a picture of human language consisting of a few referential particles operating upon a vastly larger substrate of otherwise unremarkable word-object associations. The transformative power of grammatical units is further highlighted since just one was enough to displace human codes into a more interesting region of the morphospace. This invites us to try more refined *A* matrices with grammatical particles introduced more carefully – e.g. based on how often pronouns substitute another word in real corpora. This also poses interesting questions regarding the sufficient role of grammatical units to trigger and sustain full-fledged language.

WordNet is just the most straightforward way to map human language into the model. Recent developments in neuroscience^51^ offer further opportunities to test our results and address new questions in evolutionary or developmental linguistics. Our morphospace also offers an elegant framework upon which to trace the progression, e.g., of synthetic languages^13,15,16^. Finally, our approach can help to further improve the comparative analysis between human and non-human (even non-living) signals^61^ as well as to natural and synthetic gene codes using codon-aminoacid mappings^62^.

## Methods

### Toy model and its design space

In^20^, a minimal model is introduced that links the set

4 $$S=\{{s}_{i},i=1,\ldots ,n\}$$

of available signals or “words” to the set

5 $$R=\{{r}_{j},j=1,\ldots ,m\}$$

of available objects or “meanings” existing in the world. In this model, a language is defined by a binary matrix $A\equiv \{{a}_{ij}\}$ such that *a*_*ij*_ = 1 if signal *s*_*i*_ names object *r*_*j*_, and *a*_*ij*_ = 0 otherwise. Hence, the set of all *n* × *m* binary matrices constitutes the *design space* Γ of our toy model.

Each language has a pair of costs associated to hearers or speakers. These costs can be computed from the language matrix *A*. They represent informational efforts that hearers must make to decode the meaning of a signal, or that speakers must pay to find the right name of an object. Entropies suitably encode such efforts. Following^20^, one choice is to define ${{\rm{\Omega }}}_{h}\equiv {H}_{m}(R|S)$ as the conditional entropy that weights the errors made by the hearer, namely:

6 $${H}_{m}(R|{s}_{i})=-\,\sum _{j=1}^{m}\,p({r}_{j}|{s}_{i}){lo}{{g}}_{m}p({r}_{j}|{s}_{i}),$$

7 $${H}_{m}(R|S)=\sum _{i=1}^{n}\,p({s}_{i}){H}_{m}(R|{s}_{i});$$

where *p*(*r*_*j*_|*s*_*i*_) is the probability that object *r*_*j*_ was referred to when the word *s*_*i*_ was uttered by a speaker. Such *confusing* probabilities depend on the ambiguity of the signals. We can also postulate the following effort for a speaker:

8 $${{\rm{\Omega }}}_{s}\equiv {H}_{n}(S)=-\,\sum _{i=1}^{n}\,p({s}_{i}){lo}{{g}}_{n}(p({s}_{i})),$$

where *p*(*s*_*i*_) is the frequency with which the *s*_*i*_ signal is employed given the matrix *A*. To compute *p*(*s*_*i*_) we assume that every object needs to be recalled equally often and that we choose indistinctly among synonyms for each object.

These costs

9 $$({{\rm{\Omega }}}_{h}(A),{{\rm{\Omega }}}_{s}(A))\equiv ({H}_{m}(R|S),{H}_{n}(S))$$

map each language into a 2-D plane where it can be visualized. Mapping every language we find the boundaries of our design space Γ into this plane. These costs are also the optimization targets of an MOO least-effort problem, so we often refer to the Ω_*h*_ − Ω_*s*_ plane as *target space*. Here we set up to explore the overall shape of our design space in target space, and what consequences this has for the model from an optimality viewpoint.

A first step is to find the extent of Γ in the Ω_*h*_ − Ω_*s*_ plane. The global minima of Ω_*h*_ and Ω_*s*_ delimit two of the boundaries of Γ. Let us assume that there are as many words as objects. Take the matrix associated to the minimal hearer effort, ${A}_{h}\equiv {I}_{n}$, where *I*_*n*_ denotes the *n* × *n* identity matrix so that *a*_*ij*_ = *δ*_*ij*_ (with *δ*_*ij*_ = 1 for *i* = *j* and zero otherwise, Fig. 1c). This matrix minimizes the effort for a hearer: signals are not degenerated and the hearer does not need to struggle with ambiguity. (Note that any one-to-one mapping would do – we use the identity just as an illustration). Naturally, Ω_*h*_(*A*_*h*_) = 0 while from equation 8 Ω_*s*_(*A*_*h*_) = *log*_*n*_(*m*). So *A*_*h*_ dwells on the top-left corner of the set of possible languages in target space (Fig. 2a). Consider on the other hand $A={A}_{s}\equiv \{{a}_{ij}={\delta }_{ik}\}$, where *k* is an arbitrary index $k\in [1,n]$. Here one given signal (*s*_*k*_) is used to name all existing *r*_*j*_ resulting in the minimal cost for the speaker. It follows from equations 7 and 8 that Ω_*h*_(*A*_*s*_) = 1 and Ω_*s*_(*A*_*s*_) = 0, so this matrix sits on the bottom-right corner of the Ω_*h*_ − Ω_*s*_ plane (Fig. 2a). Owing to the graph representing *A*_*s*_ (Fig. 1d) we refer to it as the star graph.

These optimal languages for one of the agents also entail the worst case for its counterpart. Hence, (for *n* = *m*) no matrices lie above Ω_*s*_ = *log*_*n*_(*m*) nor to the right of Ω_*h*_ = 1 (Fig. 2a). A language with as many signals as objects and with all of its signals completely degenerated sits on the upper right corner of the corresponding space. This is encoded by a block matrix filled with ones. For simplicity, the vertical axis in all figures of this paper has been rescaled by *log*_*m*_(*n*) so that the upper, horizontal boundary of the set is Ω_*s*_ = 1. This happens naturally if *n* = *m*, which we take often to be the case.

The only boundary left to ascertain is the one connecting *A*_*h*_ and *A*_*s*_ in the lower left region of target space. This constitutes the optimal tradeoff when trying to simultaneously minimize both Ω_*h*_ and Ω_*s*_, hence it is the Pareto front (Π_Γ_) of the multiobjective least effort language problem. It can have any shape as long as it is monotonously decreasing (notably, it does not need to be derivable nor continuous), and its shape is associated to phase transitions and critical points of the model^24,28–31^.

Prokopenko *et al*.^22,23^ computed analytically the global minimizers of equation 3. These turn out to be all matrices *A* that do not contain synonyms – i.e. which have just one 1 in each column. For those codes, using some algebra we come to the next expressions for the target functions:

10 $${{\rm{\Omega }}}_{h}\equiv {H}_{m}(R|S)={\mathrm{log}}_{m}\,(n)\,\sum _{i=1}^{n}\,\frac{{\rho }_{i}}{m}{lo}{{g}}_{n}({\rho }_{i}),$$

11 $${{\rm{\Omega }}}_{s}\equiv {H}_{n}(S)={\mathrm{log}}_{n}\,(m)-\sum _{i=1}^{n}\,\frac{{\rho }_{i}}{m}{lo}{{g}}_{n}({\rho }_{i}),$$

12 $${{\rm{\Omega }}}_{s}={\mathrm{log}}_{n}\,(m)-\frac{1}{{lo}{{g}}_{m}(n)}{{\rm{\Omega }}}_{h};$$

where *ρ*_*i*_ is the number of objects named by the *i*-th signal (see equation 17 below). Equation 12 defines a straight line in target space (Fig. 2a). It can be shown that minimizers of equation 3 are always Pareto optimal. The opposite is not necessarily true: there might be Pareto optimal solutions that do not minimize equation 3 ^24,28^. For that to be possible, the Pareto front needs to have cavities. But the curve from equation 12 connects *A*_*h*_ and *A*_*s*_ in target space barring that possibility. In this problem there cannot exist other Pareto optimal matrices other than the minimizers of equation 3. Hence equation 12 depicts the Pareto optimal manifold Π_Γ_ in target space.

Assuming *n* = *m*, Π_Γ_ becomes the straight line Ω_*s*_ = 1 − Ω_*h*_ (Fig. 2a). This implies that the global optimizers of equation 3 undergo a first order phase transition at $\lambda =1/2\equiv {\lambda }_{c}$^24,28,30^, thus confirming previous observations about the model^20,22,23^. In the literature it is also speculated that this phase transition has a critical point, but this could not be confirmed earlier. Equation 12 shows analytically that the front of this problem is a straight line. Pareto fronts which are a straight line have been linked to critical points^24,31^. The connection is equivalent to a geometric condition in thermodynamics that relates critical points to straight lines in energy-entropy diagrams^63–65^. Hence, the fact that our front is a straight line is an analytical proof that the model has a critical point. This criticality makes sense in the same way in which we talk about phase transitions for this model.

Again assuming *n* = *m*, the triangle shown in Fig. 2a contains all possible communication codes according to our model. For a modest *n* = 200 = *m* there are 2^*nm*^ = 2^40000^ possible codes. It rapidly becomes impossible to represent the whole design space of language networks. All the work reported in the Results section is based on a series of measurements taken upon languages distributed throughout the morphospace. For these to be representative we needed to generate an even sample of Γ across the Ω_*h*_ − Ω_*s*_ plane. Several strategies were tried with that aim, such as wiring objects to signals with a low probability *p*, generating a few Pareto optimal codes, the star and the one-to-one mappings, mutations and combinations of these, etc. This approach allowed to sample very small and isolated regions of the morphospace. To improve over this, we implemented a genetic algorithm. It involved a population of *N*_*s*_ = 10000 matrices with *n* = 200 = *m*. They were generated using the strategies just mentioned. The algorithm proceeded with mutation and crossover until the morphospace (the upper-right corner of a 30 × 30 grid in (Ω_*h*_,Ω_*s*_) ∈ [0, 1] × [0, 1]) was evenly covered. At each generation, the algorithm would take existing matrices and mutate or apply cross-over to them, then check if the newly generated matrices would lead to a more uniform distribution (by occupying squares of the grid with less representatives). If so, they would replace other matrices that belonged to over-represented squares of the grid.

We opted for 10000 language networks with ~20 matrices per grid square because of how costly it was to keep all matrices in the computer memory and to make calculations with them. These computational costs are already large for a mere *n* = 200 = *m*. Because Pareto optimal languages do not contain synonyms, a more sparse notation is possible for them and we can investigate more and larger matrices along the front. Some computations are also simplified for these languages (e.g. Ω_*h*_ and Ω_*s*_ are bound by equation 12). Because of this, we could perform an alternative sampling of *N*_*s*_ = 10000 Pareto optimal matrices with up to *n* = 1000 = *m*. Different stochastic mechanisms were used to seed a similar genetic algorithm that ensured an even sample of matrices along the front. While Pareto optimal matrices always included 1000 objects, some of the mechanisms to generate them would result in languages with less signals, but we can always assume that *n* = 1000 = *m* and that a lot of signals included only zeros in the corresponding positions of the *A* matrix. All measurements introduced in the next section have been properly normalized for comparison.

The fact that simple recipes to build matrices (and mutations thereof) resulted in a poor sampling of our language morphospace provides some relevant insight about how difficult it is to access most of Γ. In order to sample the whole space we needed non-trivial algorithms explicitly working to cover the whole space. If we would observe actual languages in singular regions of the morphospace, we could wonder about what evolutionary forces brought those languages there and suggest that more is needed than what simple rules offer for free.

### Measurements taken upon language networks

To explore the morphosapce we take a series of measurements upon the *A* matrices that relate to their size, network structure, or suitability as a model of actual human language. In the following we introduce these measurements in detail. The projection of these measurements back onto the morphospace are reported in the Results section.

#### Characterizing the vocabulary

While languages in our toy model consist of *n* × *m* matrices (which account for *n* signals naming *m* different meanings), not every available signal is actually used by every language. The effective vocabulary size is the amount of signals in a language that name at least one object:

13 $$L(A)\equiv \parallel \{{s}_{i},{a}_{ij}=1\,{\rm{for}}\,{\rm{some}}\,j=1,\ldots m\}\parallel .$$

We take this into account when normalizing certain properties.

We introduce a polysemy index *I*_*P*_ and a synonymy index *I*_*S*_ as:

14 $${I}_{P}=\sum _{{s}_{i}\in S}\,\frac{{lo}{{g}}_{m}({\rho }_{i})}{L},$$

15 $${I}_{S}=\sum _{{r}_{j}\in R}\frac{{lo}{{g}}_{L}({\sigma }_{j})}{m}.$$

Here *σ*_*j*_ is the number of signals associated to object *r*_*j*_:

16 $${\sigma }_{j}=\sum _{i=1}^{n}\,{a}_{ij},$$

and *ρ*_*i*_ is the number of objects associated to signal *s*_*i*_:

17 $${\rho }_{i}=\sum _{j=1}^{m}\,{a}_{ij}.$$

These indexes measure the average logarithm of *σ*_*j*_ and *ρ*_*i*_ respectively – i.e. the average information needed to decode an object given a signal (*I*_*P*_) and the averaged degeneracy of choices to name a given object (*I*_*S*_).

#### Network structure

Each language in our model defines a bipartite network. Its connectivity is given by the corresponding *A* matrix (Figs 1d–f and 3a,d). We refer to such a network as the *code graph*. We can derive two more networks from each code: one named *R*-*graph* (Fig. 3b,e) in which objects *r*_*j*_, *r*_*j*′_ ∈ *R* are connected if they are associated to one same (polysemous) signal, and another one named *S*-*graph* (Fig. 3c,f) in which signals *s*_*i*_, *s*_*i*′_ ∈ *S* are connected if they are synonymous. Because Pareto optimal codes do not contain synonyms, their bipartite code graphs consist of disconnected components in which the *i*-th signal binds together *ρ*_*i*_ objects (Fig. 3a). Consequently, each Pareto optimal *R*-graph is a set of independent, fully connected cliques (Fig. 3b) and *S*-graphs are isolated nodes (Fig. 3c).

We kept track of the set of all connected components of a network $C\equiv \{{C}_{l},l=1,\ldots ,{N}_{C}\}$ (with *N*_*C*_ the number of independent connected components) and their sizes ||*C*_*l*_||. Then

18 $${C}_{{\max }}\equiv \mathop{{\rm{\max }}}\limits_{l}\,\{\parallel {C}_{l}\parallel \}$$

gives us the size of the largest connected component. We also counted the frequency *f*(||*C*_*l*_||) with which components of a given size show up. Then the entropy of this distribution

19 $${H}_{C}=-\,\frac{1}{\parallel {N}_{C}\parallel }\,\sum _{l=1}^{{N}_{C}}\,f(\parallel {C}_{l}\parallel )\mathrm{log}(f(\parallel {C}_{l}\parallel ))$$

conveys information about how diverse the network is.

#### Complexity from codes as a semantic network

Semantic networks underlie human language in a way often exploited by psychological or performance tests that ask patients to list words of a given class (e.g. animals) by association. These have often been translated into mathematical graphs and analyzed with the tools of network theory^52,66^. Inspired by this philosophy, the graphs introduced above allow us to look at our language matrices as toy generative models of semantic networks. We explain in the next paragraphs how we built this generative model and we illustrate it below with an example.

Starting from an arbitrary signal or object we implement a random walk moving into adjacent objects or signals. We record the nodes visited, hence generating symbolic strings associated to elements ${r}_{j}\in R$ and ${s}_{i}\in S$. The network structure shall condition the frequency *f*(*r*_*j*_) and *f*(*s*_*i*_) with which different objects and signals are visited. The entropies

20 $${H}_{R}=-\,\sum _{j=1}^{m}\,f({r}_{j}){lo}{{g}}_{m}(f({r}_{j})),$$

21 $${H}_{S}=-\,\sum _{i\mathrm{=1}}^{n}\,f({s}_{i})lo{g}_{L}(f({s}_{i}\mathrm{))}.$$

will be large if *R* or *S* are evenly sampled. They will present lower values if the “semantic network” generated by our model introduces non-trivial sampling biases. Hence, here low entropy denotes interesting structure arising form our toy generative model. We also recorded 2-grams (couples of consecutive objects or signals during the random walk) and computed the corresponding entropies *H*_2*R*_ and *H*_2*S*_.

This procedure is limited to sampling from the connected component to which the first node (chosen at random) belongs. If, by chance, we would land in a small connected component, these entropies would be artificially low disregarding the structure that could exist elsewhere in the network. To avoid this situation we imposed that our generative model jumps randomly when an object was repeated twice in a row since the last random jump, or since the start of the random walk. (We also interrupted the random walk when signals, instead of objects, were repeated. Results were largely the same).

Let us follow the explicit example depicted in Supporting Fig. 1. Indexes of signals and objects are interchangeable, so they have been named as they were first sampled by the generative model. We start out by picking up a random signal (*s*_1_) from the language network from Fig. 3d. From there, we move randomly into neighboring object *r*_1_, then into neighboring signal *s*_2_, then into neighboring object *r*_2_, neighboring signal *s*_3_, and then, by chance, we bounce back into neighboring object *r*_2_. Thus far we have generated the symbolic sequence (*s*_1_, *r*_1_, *s*_2_, *r*_2_, *s*_3_, *r*_2_). Here, *r*_2_ has just been repeated twice in a row since the sampling started. This is our condition to perform a random jump to avoid getting stuck in small connected components (despite the fact that this network has only one connected component). From *r*_2_ we jump to a randomly chosen signal that happens to be *s*_4_ (dashed arrow in Supporting Fig. 1). From there objects *r*_3_ and *r*_4_ are sampled by random walk, sampling again *s*_4_ in between. As noted above, in other implementations of the generative model we decided to stop when a signal was repeated twice since the beginning of the sampling or since the last random jump. This is not the case in this example, so we proceed to sample *r*_5_, and so on.

We have produced the symbolic sequence:

$$({s}_{1},{r}_{1},{s}_{2},{r}_{2},{s}_{3},{r}_{2},{s}_{4},{r}_{3},{s}_{4},{r}_{4},{s}_{4},{r}_{5},\ldots ).$$

Actual sequences are much longer. From here we count the frequency with which each signal (*f*(*s*_*i*_)) and object (*f*(*r*_*j*_)) has been sampled and proceed to compute the entropies in equations 20 and 21. If we remove the objects from this sequence, we are left with the signal sequence: $({s}_{1},{s}_{2},{s}_{3},{s}_{4},{s}_{4},{s}_{4},\ldots )$, which contains the 2-grams $\{({s}_{1},{s}_{2}),({s}_{2},{s}_{3}),({s}_{3},{s}_{4}),({s}_{4},{s}_{4}),({s}_{4},{s}_{4}),\ldots \}$. From here we count the frequencies of digrams and compute the associated entropy *H*_2*S*_. A similar procedure is followed to produce an entropy for digrams of objects *H*_2*R*_.

#### Zipf, and other power laws

Assuming that every object needs to be recalled equally often, and that whenever an object *r*_*j*_ is recalled we choose uniformly among all the synonymous words naming *r*_*j*_; we can compute the frequency with which a word would show up given a matrix *A*. This is far from realistic: not all objects need to be recalled equally often, and not all names for an object are used indistinctly. This does not prevent numerical speculation about computational aspects of the model, which might also be informative about the richness of the morphospace. In any case, this has been the strategy used in the literature to compute the frequency with which each word is retrieved by the model^20,22,23^.

The word frequency distribution of a language follows a power law if the *i*-th more frequent signal appears with frequency *P*(*s*_*i*_) ~ 1/*i*^*γ*^. This distribution becomes Zipf’s law if *γ* = 1, such that the second most frequent signal appears half the times of the first one, the third most frequent signal appears a third of the first one, etc. Once we had built word frequency distributions for each language network, we followed the prescriptions in^67^ to evaluate how well each of these distributions were explained by power laws. In one approach, we used a Kolmogorov-Smirnov (KS) test to compare word distributions from the model to Zipf’s law – no fitting of the original distribution was performed here, since Zipf’s was an Ansatz. In another approach, we fitted our model distributions to power laws with arbitrary exponents – i.e. we did not impose *γ* = 1. We then used another KS-test to assess the fitness of the original distributions to this generalized power laws^12,45^.

### Data availability

This is a mostly theoretical work based on computational experiments. Instructions have been provided which allow a reader to reproduce our work. The empirical data used in this paper has been obtained from the WordNet database and is freely available online.

## Electronic supplementary material

Appendix A

Supporting figure
