# Supplementary material for: The morphospace of language networks
Source: Sci Rep. 2018 Jul 11;8:10465. doi: 10.1038/s41598-018-28820-0 (PMC6041342; doi:10.1038/s41598-018-28820-0)
Supplement: Supplementary file 1 — Appendix A [file 41598_2018_28820_MOESM1_ESM.pdf]

The morphospace of language networks

Luís F Seoane[1,*] **and Ricard Solé**[2, 3, 4, *]

[1]Department of Physics, Massachusetts Institute of Technology, 02139, USA
[2]ICREA-Complex Systems Lab, Universitat Pompeu Fabra, 08003, Barcelona
[3]Institut de Biologia Evolutiva (CSIC-UPF), 08003 Barcelona
[4]Santa Fe Institute, 399 Hyde Park Road, Santa Fe NM 87501, USA
[*]seoane@mit.edu, ricard.sole@upf.edu

ABSTRACT

This file contains an appendix with supporting material of the main text. It reports the different measurements taken upon Pareto optimal languages.

A Complexity of language networks along the Pareto front

In the Results section we reported a series of measurements taken over an even sample across the morphospace. Those results are complemented here by measurements taken over a more exhaustive sample of the Pareto front which includes larger matrices (with up to 1 000 signals and objects, as opposed to the $n = 200 = m$ in the main text). In the following sections we analyze the same measurements of vocabulary, network structure, matrix as a generative model, and goodness of fit to power-law that we analyzed above.

The Pareto optimal manifold is just a straight line, which allows us to present simpler plots. We argued in the Methods section that straight lines correspond to critical points if the model is studied from a StatMech perspective[16,17], hence we often refer to the Pareto front as the critical point or critical manifold indistinctly. In all plots below, the horizontal axis reports the value of $\Omega_h \equiv H(R|S)$ along the front. This is, the one-to-one mapping lies at the leftmost part of the plot and the star graph at the rightmost end.

A.1 Characterizing the vocabulary

By definition, Pareto optimal languages have no synonyms hence $I_S = 0$. We report next vocabulary size (L) and polysemy index (I_P) along the front.

Figure S1 shows that the effective vocabulary size does not decrease linearly as we proceed from the one-to-one mapping ($L = n$) to the star ($L = 1$). Furthermore, at most given points along the front, there seem to be several languages with the same effort for both speaker and hearers, and yet with different vocabulary sizes. This indicates that there are different strategies to achieve the same degree of optimality, or that being Pareto optimal leaves the diversity of languages largely unconstrained.

Regarding polysemy, we could also expect that it would build up uniformly as we approach the star code because words take up more and mode meanings as they depart from the one-to-one mapping. Instead we see that at each point along the front there are very different codes showing a range of polysemy (figure S1, inset). The maximum of this range does grow with $H_m(R|S)$. Eventually, I_P has to be maximum and unique for the star graph ($I_P = 1$, since a single signal is maximally polysemic). The fact that similar Pareto optimal codes present such diverse I_P (as well as L), even close to the star graph, suggests a great diversity within the critical point of the model. We will find that this is a recurrent theme of Pareto optimal languages for other measurements as well.

A.2 Network structure

We recall now the bipartite network structure (code graph) and the corresponding R- and S-graphs in object and signal space. These are naturally induced by the A matrices as illustrated in figure 3 in the main text Associated to them, we report the size of the largest connected component C_{max} for each graph, and the entropy of the distribution of component sizes (H_C) as introduced in the Methods section.

For languages along the Pareto front, the largest connected component of the S-graph has trivially just 1 signal (because, again, there are no synonyms). This implies that the largest connected component of the code and R-graphs are virtually the same. Figure S2**a** shows the size (normalized to the maximum value possible) of the largest component of the code graph along

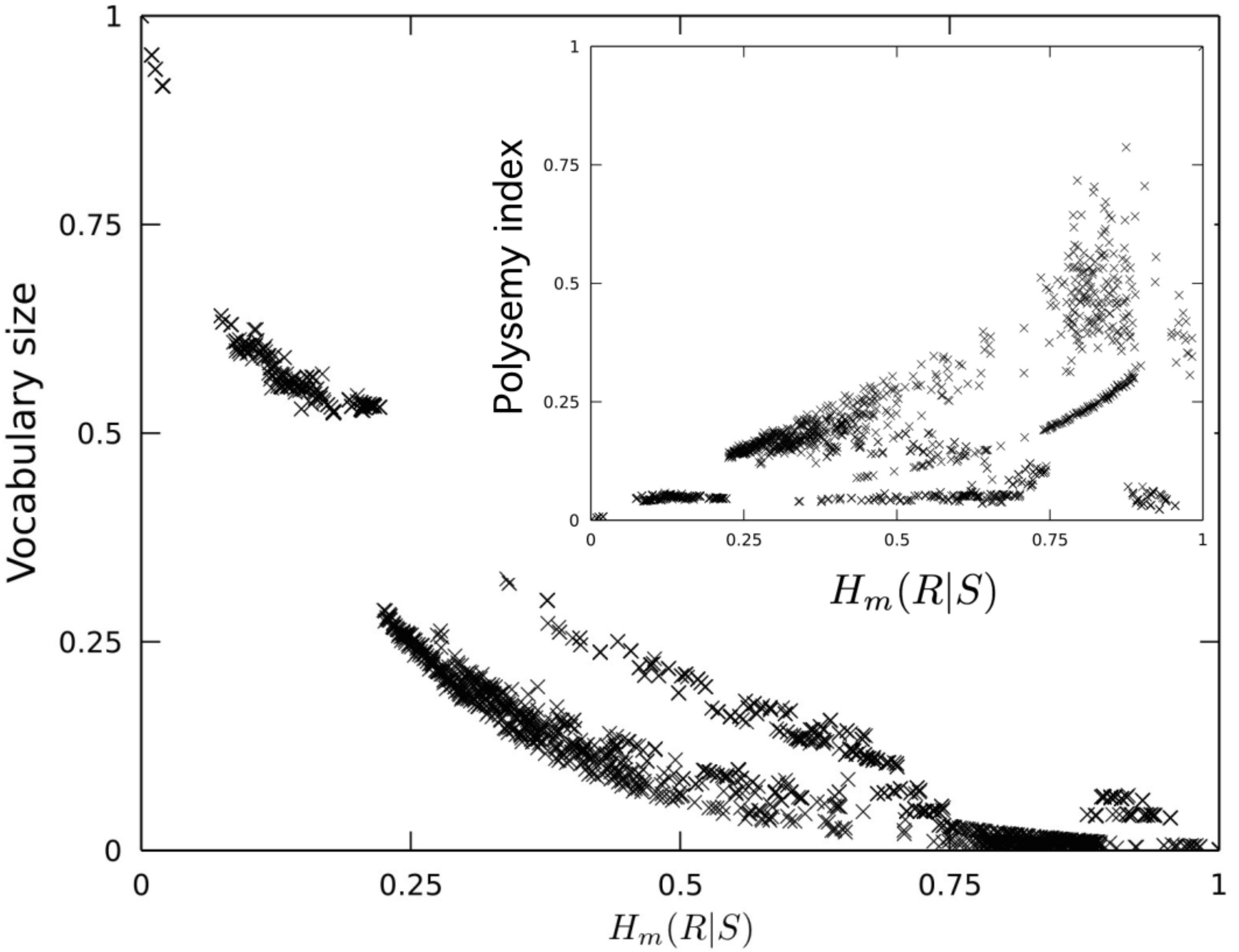

Supplementary Fig. S1. Vocabulary size and polysemy along the Pareto front. a Codes along the Pareto front keep a relatively low vocabulary except close to the one-to-one mapping. Also, two branches seem noticeable around the middle of the front, suggesting that similar Pareto optimal values of $H_m(R|S)$ and of $H_n(S)$ can be achieved with differently wired codes. **b** A reduced vocabulary size does not result in a strictly monotonous increase of polysemy as we approach the star code. Instead, languages with similar $H_m(R|S)$ may present different polysemy levels. The range available grows as we approach the maximally ambiguous code.

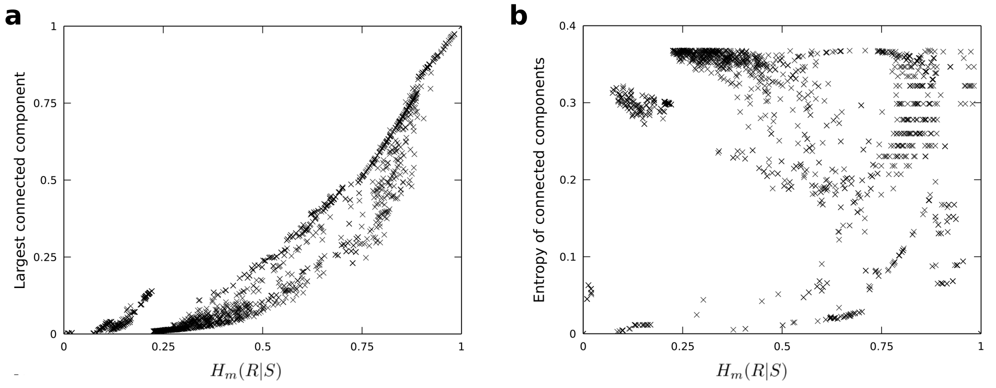

Supplementary Fig. S2. **Network connectivity along the Pareto front. a** Along the front, the size of the largest connected component grows from $1/m$ to 1 as we move from the one-to-one mapping to the start graph. **b** The entropy of component size distribution shows a large degree of degeneracy even for single points along the front.

the front. It grows as we move from the one-to-one mapping to the star code, but this growth is again mostly non-linear and often several possibilities coexist at each point of the front.

Regarding H_C, we find no consistent pattern throughout the front (figure S2**b**). This is the measure for which we find less correlation along any direction in Pareto optimal codes, again suggesting that the diversity of networks along the front is largely unconstrained. Notwithstanding, this variability is perhaps not so salient: H_C here is small as in most of the morphospace (compare the scale in the color bar of panel 4**d** of the main text against the vertical axis of panel S2**b**). Moving apart from the star graph, we know that several signals are involved in Pareto optimal languages (as the vocabulary size implies – figure S1) and yet H_C is kept low and relatively constant throughout. This suggests that, while a lot of disconnected components coexist to make up a Pareto optimal language network, their sizes are similar resulting in just a few graphs similar to each other.

A.3 Complexity from codes as a semantic network

We turn our attention now to language matrices as generative toy models of semantic relationships. Therefore, we had introduced a random walk over code graphs in Methods. These allowed us to capture, with a series of entropies ($H_{R,S}$ and $H_{2R,2S}$), whether the network structure somehow biased the sampling of signals or objects as it traversed the network randomly. Large entropies in the distribution of sampled objects or signals implied networks that do not induce remarkable structures. Meanwhile, noteworthy biases in object or signal sampling would result in lower entropies than expected.

By construction, H_R must be maximum at both extremes of the front and non-trivial along it (figure S3**a**). In the one-to-one mapping, a same object is always sampled repeatedly, resulting in a continuous reset of the random walk process as described in Methods. Because the starting point is uniformly random, so must be the random walk and H_R collapses to 1. This results in a maximal entropy over signals as well (figure S3**b**). At the star graph, only one signal produces a valid sample of the code graph, and again this sample is uniform over objects (resulting in $H_R = 1$, figure S3**a**) but this implies a maximally asymmetric sampling of words ($H_S = 0$, figure S3**b**). Along the front, objects group up in clusters of different size, resulting in potentially greater biases towards some objects than others. This results in the possibility of a lower H_R, which is not always fulfilled. As in other cases, we see that a same point along the front hosts several different language networks with diverse H_R values. The set of languages that produce a more remarkable structure (i.e. lowest H_R) is very close to the star graph (figure S3**a**). Overall, H_R is large along the Pareto front as it was throughout the morphospace. The number of objects that a word links together determines how often that signal can be sampled through the random walker without reseting the process. This results in a smooth curve of decreasing entropy for H_S (figure S3**b**). This suggests an explanation for the area of the morphospace with lowest H_S in figure 5**b** of the main text, near the star graph.

The entropy of 2-gram objects also has to be maximal at both ends of the front (figure S3**c**). It remains largely unconstrained along the rest of the front, with little correlation and again large variability at any given point. The entropy of 2-gram signals again decays to 0 as the start graph is approached, but the decay is now less smooth and the range of values of H_{2S} at any given point is larger than for H_S.

A.4 Zipf, and other power laws

We computed the goodness of fit of word distribution to either Zipf or power-laws with arbitrary exponents. One of the caveats is that our languages across the morphospace are relatively small ($n = 200 = m$). While this is partly alleviated here (thanks

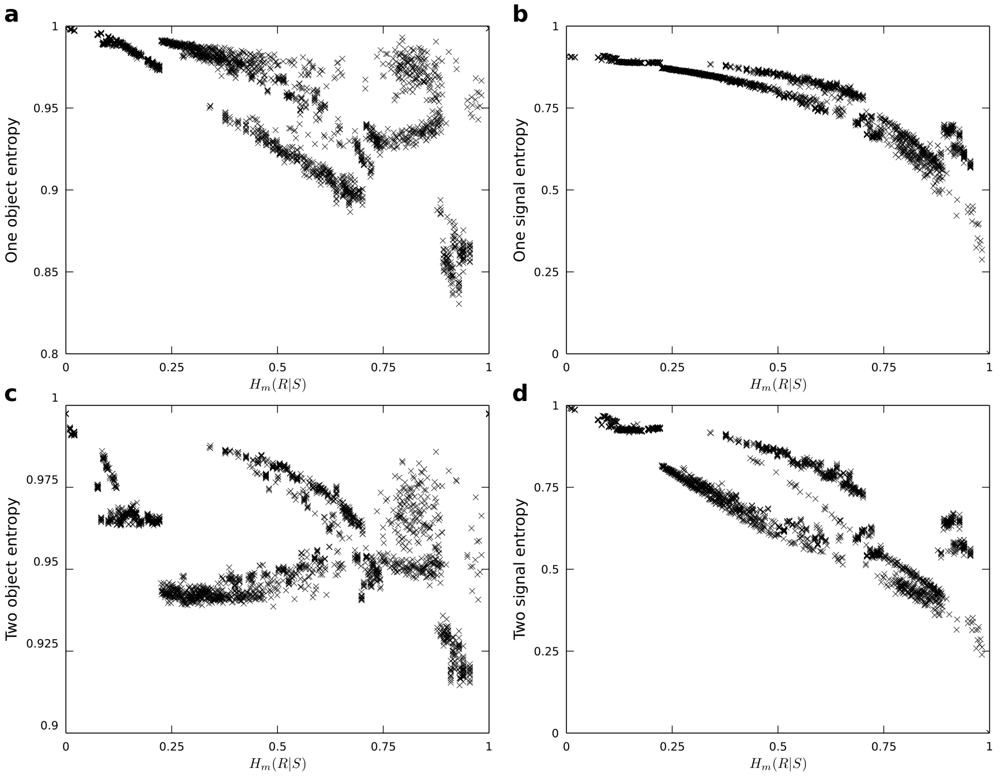

Supplementary Fig. S3. Complexity of codes as a random generative model along the Pareto front. a The entropy of objects as sampled by a random walker (H_R) over the language network is maximal at either end of the front and presents a minimum close to the star graph. **b** The entropy of objects as sampled by a random walker (H_S) decreases rather smoothly along the Pareto front as we move from the one-to-one mapping to the star. **c** The entropy of 2-gram objects as sampled by a random walker (H_{2R}) presents less structure than H_R and is still maximal at either extreme. **d** The entropy of 2-grams signals as sampled by a random walker (H_{2S}) also decreases as we move along the front, but in a less structured fashion.

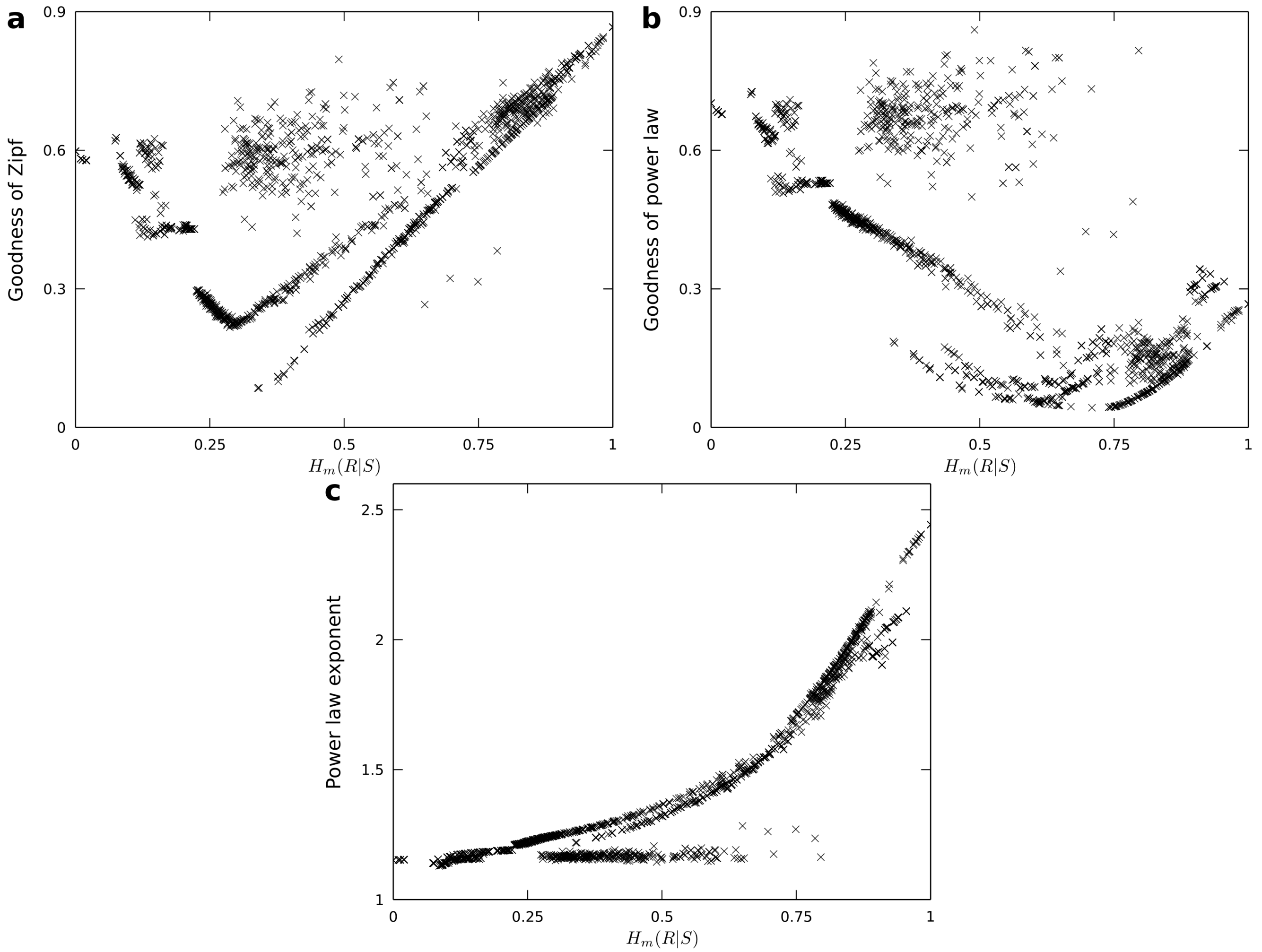

Supplementary Fig. S4. Power laws from the least-effort model along the Pareto front. a Goodness of fit of the word distribution from the toy, least-effort model to a Zipf law along the Pareto front. **b** Goodness of fit of the word distribution from the model to an arbitrary power law along the Pareto front. **c** Exponent obtained along the Pareto front when fitting the word distribution of the model to the arbitrary power law from panel **b**.

to languages with up to 1 000 signals), these are nevertheless meager numbers. The results in this section can again mount evidence against the least-effort hypothesis as the origin of Zipf's distribution in human language, but this must be taken with extreme care given the computational shortages just mentioned and also considering the limitations of the model.

Regarding goodness of fit to Zipf, along the Pareto front we find again a great variety of codes even within single points (figure S4**a**). This indicates, as pointed out above and already anticipated in[?, 9], that least-effort alone would not be enough to enforce Zipf's distribution into word corpora – at least not within this very limited toy model. There is a clear minimum of KS-score (i.e. maximum fitness to Zipf's distribution, figure S4**a**) around $\Omega_h \sim 0.3$ (hence $\Omega_s \sim 0.7$). This is close to, but not right at the value $\Omega_h = 1/2 = \Omega_s$ put forward by[21] for theoretical reasons. Also, the minimum KS-score (~ 0.1) is larger than scores reached deeper inside the morphospace (figure 6**a** in the main text). According to this, the observation of Zipf's law in natural corpora would be evidence against the least-effort principles captured by the model.

Regarding the goodness of fit to arbitrary power laws (figure S4**b**), we find a more shallow minimum suggesting a broader region of interesting Pareto optimal languages. Looking at the exponents that come out of those fits (figure S4**c**), we find two branches as we move in the direction of increasing $H_m(R|S)$ (i.e. towards the star graph): i) a branch of roughly constant and low exponents close to 1 (hence similar to Zipf's law), ii) a branch of exponents that increase monotonously with $H_m(R|S)$. It is difficult to asses which of these branches is yielding the lowest KS-score (best fit) in figure S4**b**.

A.5 Code archetypes along the Pareto front

Finally, as we did for the whole language morphospace, we analyzed possible archetypes clustering out of the measurements across the Pareto front. We moved into PC space and tried building 3 and 5 language archetypes using k-means clustering. For $k = 3$ we found three relatively stable clusters: i) a few codes near the one-to-one graph, ii) a few others near the star network, and iii) all remaining codes along the front. However, the boundaries between the clusters changed notably after different initializations of the algorithm, sometimes leaving the third group almost without elements. With $k = 5$, the clusters found were not stable at all, meaning that different instantiations of k-means would lump codes together in very different ways. Those clusters would also overlap when plotted along the Pareto front.

These results are very unlike the outcome for the whole morphospace. There, applying k-means several times with random initializations would consistently yield the same broad classes, which were clearly segregated across the morphospace with little overlap at their borders. Our inability to converge into well defined archetypes at the Pareto front is yet another indication of its huge diversity. We should also be careful about the previous clustering of Pareto optima within groups I and V (see Results section in the main text). Fortunately, those classes reach deeper inside the morphospace and do not seem to depend so much on Pareto optimal solutions.

References

1. Bickerton, D. *Language and species*. (University of Chicago Press, 1992).
2. Szathmáry, E. & Maynard Smith, J. *Major Transitions in Evolution*. (Oxford: Oxford University Press, 1997).
3. Deacon, T.W. *The symbolic species: The co-evolution of language and the brain*. (WW Norton & Company, 1998).
4. Bickerton, D. *More than nature needs: Language, mind, and evolution*. (Harvard University Press, 2014).
5. Berwick, R.C. & Chomsky N. *Why Only Us: Language and Evolution*. (MIT Press, 2015).
6. Suddendorf, T. *The gap: The science of what separates us from other animals*. (Basic Books, 2013).
7. Jablonka, E. & Szathmáry, E. The evolution of information storage and heredity. *Trends Ecol. Evol.* **10**(5), 206-211 (1995).
8. Jablonka, E. & Lamb, M.J. The evolution of information in the major transitions. *J. Theor. Biol.* **239**(2), 236-246 (2006).
9. Prokopenko, M., Ay, N., Obst, O. & Polani, D. Phase transitions in least-effort communications. *J. Stat. Mech.* **11**, P11025 (2010).
10. Salge, C, Ay, N., Polani, D. & Prokopenko, M. Zipf's law: balancing signal usage cost and communication efficiency. PLoS one **10**(10), e0139475 (2015).
11. Ferrer i Cancho, R. & Solé, R.V. Least effort and the origins of scaling in human language. *Proc. Natl. Acad. Sci.* **100**(3), 788-791 (2003).
12. Solé, R.V. & Seoane, L.F. Ambiguity in Language Networks. *Linguist. Rev.* **32**(1), 5-35 (2014).
13. Seoane, L.F. & Solé, R. A multiobjective optimization approach to statistical mechanics. Preprint at https://arxiv.org/abs/1310.6372 (2013).
14. Seoane, L.F. & Solé, R. Phase transitions in Pareto optimal complex networks. *Phys. Rev. E* **92**(3), 032807 (2015).
15. Seoane, L.F. & Solé, R. Multiobjective optimization and phase transitions. *Springer Proceedings in Complexity*, 259-270 (2015).
16. Seoane, L.F. & Solé, R. Systems poised to criticality through Pareto selective forces. Preprint at https://arxiv.org/abs/1510.08697 (2015).
17. Seoane, L.F. *Multiobjective Optimization in Models of Synthetic and Natural Living Systems*. PhD dissertation, Universitat Pompeu Fabra, Department of Experimental and Health Sciences, May (2016).
18. Huth, A.G., Nishimoto, S., Vu, A.T. & Gallant, J.L. A continuous semantic space describes the representation of thousands of object and action categories across the human brain. *Neuron* **76**(6), 1210-1224 (2012).
19. Clauset, A., Shalizi, C.R. & Newman, M.E. Power-law distributions in empirical data. *SIAM Rev.* **51**(4), 661-703 (2009).
20. Zipf, G.K. *Human Behavior and the Principle of Least Effort*. (Addison-Wesley, Reading, MA, 1949).
21. Fortuny, J. & Corominas-Murtra, B. On the origin of ambiguity in efficient communication. *J. Logic Lang. Inform.* **22**(3), 249-267 (2013).
22. Corominas-Murtra, B., Hanel, R. & Thurner, S. Understanding scaling through history-dependent processes with collapsing sample space. *Proc. Natl. Acad. Sci.* 112(17), 5348-5353 (2015).

23. Steyvers, M. & Tenenbaum, J.B. The Large-scale structure of semantic networks: Statistical analyses and a model of semantic growth. *Cognitive science* **29**(1), 41-78 (2005).

24. Corominas-Murtra, B. & Solé, R.V. Universality of Zipf's law. *Phys. Rev. E* **82**(1), 011102 (2010).

25. Corominas-Murtra, B., Fortuny, J. & Solé, R.V. Emergence of Zipf's law in the evolution of communication. *Phys. Rev. E* **83**(3), 036115 (2011).

26. Ferrer i Cancho, R. The variation of Zipf's law in human language. *Eur. Phys. J. B* **44**(2), 249-257 (2005).

27. Baixeries, J., Elvevåg, B. & Ferrer i Cancho, R. The evolution of the exponent of Zipf's law in language ontogeny. *PLoS one* **8**(3), p.e53227 (2013).

28. Lloyd, S.P. Least squares quantization in PCM. *IEEE Trans. Inf. Theory* **28**(2), 129-137 (1982).

29. Miller, G.A. WordNet: a lexical database for English. *Commun. ACM* **38**(11), 39-41 (1995).

30. Fellbaum, C., ed. WordNet: An Electronic Lexical Database. (Cambridge, MA: MIT Press, 1998).

31. Nowak, M.A., Plotkin, J.B. & Krakauer, D.C. The evolutionary language game. *J. Theor. Biol.* **200**(2), 147-162 (1999).

32. Nowak, M.A., Komarova, N.L. & Niyogi, P. Computational and evolutionary aspects of language. *Nature* **417**, 611-617 (2002).

33. Nowak, M.A. & Krakauer, D.C. The evolution of language. *Proc. Natl. Acad. Sci. USA.* **96**, 8028-8033 (1999).

34. Deb, K. *Multi-objective optimization using evolutionary algorithms*. (Wiley, New Dehli, 2003).

35. Coello, C.C. Evolutionary multi-objective optimization: a historical view of the field. *IEEE Comput. Intel. M.* **1**(1), 28-36 (2006).

36. Schuster, P. Optimization of multiple criteria: Pareto efficiency and fast heuristics should be more popular than they are. *Complexity* **18**, 5-7 (2012).

37. Solé, R. Language networks; their structure, function and evolution. *Complexity* **15**, 20-26 (2010).

38. Ferrer i Cancho, R., Koehler, R. & Solé, R. Patterns in syntactic dependency networks. *Phys. Rev. E* **69**, 32767 (2004).

39. Ferrer i Cancho, R. When language breaks into pieces: A conflict between communication through isolated signals and language.. *Biosystems* **84**, 242-253 (2006).

40. Ferrer i Cancho, R., Bollobás, B. & Riordan, O. The consequences of Zipf?s law for syntax and symbolic reference. *Proc R Soc Lond Ser B* **272**, 561-565 (2005).

41. Solé, R. Syntax for free? *Nature* **434**, 289 (2005).

42. Jaeger, T.F. & Levy, R.P. Speakers optimize information density through syntactic reduction. *Adv. Neur. In.*, 849-856 (2006).

43. Frank, A. & Jaeger, T.F. Speaking rationally: Uniform information density as an optimal strategy for language production. In *Proceedings of the 30th annual meeting of the cognitive science society*, 933-938. (Washington, DC: Cognitive Science Society, 2008).

44. Jaeger, T.F. Redundancy and reduction: Speakers manage syntactic information density. *Cognitive Psychol.* **61**(1), 23-62 (2010).

45. Piantadosi, S.T., Tily, H. & Gibson, E. Word lengths are optimized for efficient communication. *Proc. Nat. Acad. Sci.* **108**(9), 3526-3529 (2011).

46. Mahowald, K., Fedorenko, E., Piantadosi, S.T. & Gibson, E. Speakers choose shorter words in predictive contexts. *Cognition* **126**(2), 313-318 (2013).

47. Shannon, C.E. A Mathematical Theory of Communication. *Bell System Technical Journal* **27**(3), 37-423 (1948).

48. Kirby, S. Spontaneous evolution of linguistic structure-an iterated learning model of the emergence of regularity and irregularity. *IEEE T. Evolut. Comput.* **5**(2), 102-110 (2001).

49. Kirby, S. Natural language from artificial life. *Artif. Life* **8**(2), 185-215 (2002).

50. Kirby, S., Cornish, H. & Smith, K. Cumulative cultural evolution in the laboratory: An experimental approach to the origins of structure in human language. *Proc. Nat. Acad. Sci.* **105**(31), 10681-10686 (2008).

51. Steels, L. *The talking heads experiment: Origins of words and meanings*. (Language Science Press, 2015).

52. Steels, L. *The synthetic modeling of language origins*. Evol. Comm. **1**, 1-34 (1997).

53. Cangelosi, A. & Parisi, D. The emergence of a 'language' in an evolving population of neural networks. *Connect. Sci.* **10**(2), 83-97 (1998).

54. Huth, A.G., de Heer, W.A., Griffiths, T.L., Theunissen, F.E. & Gallant, J.L. Natural speech reveals the semantic maps that tile human cerebral cortex. *Nature* **532**(7600), 453-458 (2016).

55. Cover, T.H. & Thomas, J.A. *Elements of Information Theory*. (John Wiley, New York, 1991).

56. McGhee, G.R. *Theoretical morphology. The concept and its application*. (Columbia U. Press, 1999).

57. Niklas, K.J. *The evolutionary biology of plants*. (Chicago U. Press, 1997).

58. Niklas, K.J. Computer models of early land plant evolution. *Annu. Rev. Earth Planet. Sci.* **32**, 47-66 (2004).

59. Raup, D. Geometric analysis of shell coiling: general problems. Paleobiology **40**, 1178-1190 (1965).

60. Avena-Koenigsberger, A., Goni, J., Solé, R. & Sporns, O. Network morphospace. J. R. Soc. Interface **12**, 20140881 (2015).

61. Christiansen, M.H., Chater, N. & Culicover, P.W. *Creating language: Integrating evolution, acquisition, and processing*. (MIT Press, 2016).

62. Corominas-Murtra, B., Seoane, L.F. & Solé, R. Zipf's law, unbounded complexity and open-ended evolution. Preprint at https://arxiv.org/pdf/1612.01605.pdf (2016).

63. Mora, T. & Bialek, W. Are biological systems poised at criticality? *J. Stat. Phys.* **144**(2), 268-302 (2011).

64. Tkačik, G., Marre, O., Mora, T., Amodei, D., Berry II, M.J. & Bialek, W. The simplest maximum entropy model for collective behavior in a neural network. *J. Stat. Mech.* **2013**(03), P03011 (2013).

65. Tkačik, G., Mora, T., Marre, O., Amodei, D., Palmer, S.E., Berry, M.J. & Bialek, W. Thermodynamics and signatures of criticality in a network of neurons. *Proc. Nat. Acad. Sci.* **112**(37), 11508-11513 (2015).

66. Goñi, J., Arrondo, G., Sepulcre, J., Martincorena, I., de Mendizábal, N.V., Corominas-Murtra, B., Bejarano, B., Ardanza-Trevijano, S., Peraita, H., Wall, D.P. & Villoslada, P. The semantic organization of the animal category: evidence from semantic verbal fluency and network theory. *Cogn. Process.* **12**(2), 183-196 (2011).

67. Doyle, L.R., McCowan, B., Johnston, S. & Hanser, S.F. Information theory, animal communication, and the search for extraterrestrial intelligence. *Acta Astronautica* **68**(3-4), 406-417 (2011).

68. . Tlusty, T. A model for the emergence of the genetic code as a transition in a noisy information channel. *J. Theor. Biol.* **249**, 331-342 (2007).
