# Supplementary figures and images for: The morphospace of language networks
Source: Sci Rep. 2018 Jul 11;8:10465. doi: 10.1038/s41598-018-28820-0 (PMC6041342; doi:10.1038/s41598-018-28820-0)

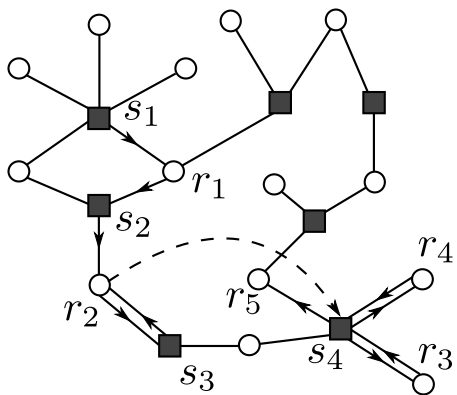

Supplement: Supplementary file 2 — Supporting figure [file 41598_2018_28820_MOESM2_ESM.pdf]
